# Ethics of cost analyses in medical education

**DOI:** 10.1007/s40037-013-0064-1

**Published:** 2013-06-14

**Authors:** Kieran Walsh

**Affiliations:** BMJ Learning, BMJ Group, BMA House, Tavistock Sq., London, WC1H 9JR UK

**Keywords:** Cost, Value, Medical education, Distributive justice

## Abstract

Cost analyses in medical education are rarely straightforward, and rarely lead to clear-cut conclusions. Occasionally they do lead to clear conclusions but even when that happens, some stakeholders will ask difficult but valid questions about what to do following cost analyses–specifically about distributive justice in the allocation of resources. At present there are few or no debates about these issues and rationing decisions that are taken in medical education are largely made subconsciously. Distributive justice ‘concerns the nature of a socially just allocation of goods in a society’. Inevitably there is a large degree of subjectivity in the judgment as to whether an allocation is seen as socially just or ethical. There are different principles by which we can view distributive justice and which therefore affect the prism of subjectivity through which we see certain problems. For example, we might say that distributive justice at a certain institution or in a certain medical education system operates according to the principle that resources must be divided equally amongst learners. Another system may say that resources should be distributed according to the needs of learners or even of patients. No ethical system or model is inherently right or wrong, they depend on the context in which the educator is working.

‘In the current, cost-constrained environment, those funding the education of our doctors will no longer tolerate an approach of quality at any cost'.

Liam Donaldson [1]

On first impressions it would seem that cost analyses in medical education are a fairly straightforward activity. A medical education intervention (for example a simulation programme) might have lower costs and be associated with higher effectiveness or more benefits or utility than alternatives [2]. The argument would seem to end there; such an intervention would surely be a natural first choice in medical education curricula. However, cost analyses are rarely so straightforward and rarely lead to clear-cut conclusions. Occasionally they do lead to clear conclusions but even when that happens, some stakeholders will ask difficult but valid questions about what to do following cost analyses–specifically about distributive justice in the allocation of resources [3]. These and other questions must be answered. The questions are relevant to systems of medical education in all countries.

Distributive justice is sometimes best understood through case examples. Say a medical school runs two programmes concurrently. One programme has been set up to improve communication skills among graduate doctors who struggle with this area of competence. Another programme has been set up to improve interdisciplinary team working among all newly graduated health care professionals. Both programmes turn out to be a success. Both are associated with relatively modest costs. The communication skills programme significantly improves the communication skills of the bottom 10 % of graduates. Patient feedback to those doctors has significantly improved. The team working programme improves team working at the local institution where the junior doctors, nurses and allied health care professionals all work. The multisource feedback system amongst health care professionals has picked up qualitative improvements, even though patient satisfaction has not altered. However, 1 year on, there have been cuts to the budget and the school’s leaders must decide which of the two programmes to continue. One programme has tangible outcomes, albeit amongst a small minority of doctors. The other programme has affected larger numbers of health care professionals, even though its outcomes have been more subtle. On the basis of these analyses, which of the two programmes should be continued? The answer is not straightforward; nor is it obvious whose opinions should be sought. Should educators, learners, patients or even medical education economists decide? The few doctors whose communication skills have improved would probably favour their programme; equally the many interdisciplinary team members may favour theirs. As analysts who have looked at such issues when deciding upon the allocation of health care resources have found, answers to these questions are not straightforward [4].

Some opinions may even be divisive–some may ask why scarce resources should be spent on a few people with poor communication skills and may even blame the poor communicators for not developing their own skills at the rate that their own colleagues have done. Some debates about what should be costed will be even more difficult. Should funding be spent on programmes that will produce tertiary care oncologists who might be directly responsible for saving lives or on programmes to widen participation in medicine which will have longer term albeit less quantitatively measurable outcomes? At present part of the problem is that there are no debates about such questions and little evidence on which to base judgements, and so rationing decisions that are taken in medical education are largely made subconsciously (12). How are we to change medical education so that we can make ethical decisions in this regard more conscious ones and to ensure that we have an open debate about what decisions we should take and how we should take these decisions?

It is likely that we will do so by following in the footsteps of those who have had to make similar difficult decisions regarding the delivery of health care and examine how the concept of distributive justice could help us. Distributive justice ‘concerns the nature of a socially just allocation of goods in a society’ [5]. Inevitably there is a large degree of subjectivity in the judgment as to whether an allocation is seen as socially just or ethical. There are different principles by which we can view distributive justice and which therefore affect the prism of subjectivity through which we see certain problems. For example, we might say that distributive justice at a certain institution or in a certain medical education system operates according to the principle that resources must be divided equally amongst learners. Another system may say that resources should be distributed according to the needs of learners or even of patients [6].

It is easy to see how these different principles would result in quite different outcomes. In the above example funding the medical education intervention to remediate communication skills among poorly performing graduates would be an example of prioritising funding according to the needs of learners. By contrast, the interdisciplinary education intervention will spread funding equally among all health care professionals regardless of their needs and so is an example of equal distribution of resources among learners. Neither funding model is right or wrong, it depends on the context and what you are trying to achieve. Another level of complexity will be added when we look at the funding model that the system of medical education is based on. In some countries, medical education is free to students; in others students have to pay tuition fees [7]. When a student is paying a fee, should they have a greater say in the allocation of the resources that will create the medical education that they will receive? It is by no means certain that they should, but even accepting for the point of argument that they should, how will we capture learner preferences and rights in cost analytic models that use aggregate data?

Some people will feel that it is impossible to apply cost analytic methods to medical education or that even if you do, the process itself is inherently unethical, insofar as it reduces medical education to meaningless numbers. The medical education community may respect this view but it is likely that budget holders at a macroeconomic level will pay much heed to it. Others might argue that we are better off ‘muddling through’ than by trying too hard to allocate resources to all initiatives in a micro-economic fashion [8]. This outlook is not an unreasonable one and for some contexts in medical education it might be the right one. However, as with all the decisions or approaches taken, they should be taken explicitly and by consensus among relevant stakeholders.

## Conclusions

Distributive justice ‘concerns the nature of a socially just allocation of goods in a society’ [5]. Medical educators might decide to allocate resources equally amongst learners, might allocate more to learners with greater needs, or might allocate resources according to population health needs. No model is inherently right or wrong, it depends on the context in which it is being used. When making decisions about how to allocate resources, medical educators should consciously think through how to make decisions that will be fair to a wide range of stakeholders. If they decide to allocate more resources to one group, they should make this explicit, and should make their reasoning behind decisions explicit too.
